# Life barcoded by DNA barcodes

**DOI:** 10.1007/s12686-022-01291-2

**Published:** 2022-08-15

**Authors:** Mali Guo, Chaohai Yuan, Leyan Tao, Yafei Cai, Wei Zhang

**Affiliations:** 1 College of Animal Science and Technology, Nanjing Agricultural University, Nanjing, 210095 China; 2 National Experimental Teaching Demonstration Center of Animal Science, Nanjing Agricultural University, Nanjing, 210095 China

**Keywords:** DNA barcode, *COI*, Reference libraries, Mini-barcode, DNA metabarcoding

## Abstract

The modern concept of DNA-based barcoding for cataloguing biodiversity was proposed in 2003 by first adopting an approximately 600 bp fragment of the mitochondrial *COI* gene to compare via nucleotide alignments with known sequences from specimens previously identified by taxonomists. Other standardized regions meeting barcoding criteria then are also evolving as DNA barcodes for fast, reliable and inexpensive assessment of species composition across all forms of life, including animals, plants, fungi, bacteria and other microorganisms. Consequently, global DNA barcoding campaigns have resulted in the formation of many online workbenches and databases, such as BOLD system, as barcode references, and facilitated the development of mini-barcodes and metabarcoding strategies as important extensions of barcode techniques. Here we intend to give an overview of the characteristics and features of these barcode markers and major reference libraries existing for barcoding the planet’s life, as well as to address the limitations and opportunities of DNA barcodes to an increasingly broader community of science and society.

## Introduction

**Fig. 1:**
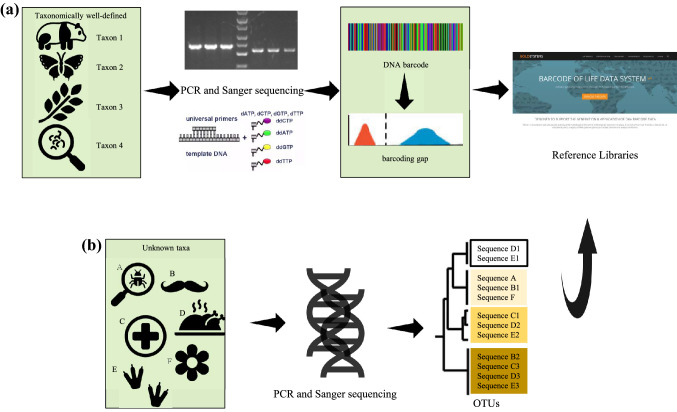
Basic workflow for getting barcode markers using Sanger sequencing. **a** Workflow for generating reference databases. **b** Workflow for taxonomic assignment of unknown samples by comparing barcode sequences with reference databases

In comparison to the universal bar code consisting of a series of vertical bars that are printed on commercial products, a DNA barcode, in a broad sense, refers to any DNA sequence adopted to identify species at any taxonomic level. To be more specific, a DNA barcode is one or more short gene sequences (generally 200–900 base pairs) taken from a standardized portion of the genome to aid species identification and discovery by employing sequence divergence based on nucleotide alignment (Emerson et al. 2011; Hebert et al. 2003a, 2004). Thus, the fundamental function of this genetic tool seeks to compare barcode sequences to reference databases to efficiently and effectively assign any biological sample to its species regardless of the visual classification of the sample (Fig. 1).

In fact, the history of genetic sequences applied in taxonomy research can be traced back to 1969 when Bicknell and Douglas found that the arrangement of species in yeast dependent on ribosomal RNA homologies in most cases agreed with the established taxonomic groupings via traditional measures (Bicknell and Douglas 1970). Before that, 250 years have been spent to catalogue about 1.2 million species by traditional taxonomic approaches through manual characterizations incorporating morphological features, which apparently is challenging and unrealistic when it is forecasted that around 7.52 million terrestrial species and 2.01 million species in the ocean still await description (Leray and Knowlton 2016; Scheffers et al. 2012). The advent of techniques for gene isolation, cloning and Sanger sequencing in the second half of last century allows the term “DNA barcode” to be first used in 1993 when length information of tandemly repeated DNA sequences from hypervariable alleles was barcoded to discriminate isolates of *Plasmodium falciparum* (Arnot et al. 1993). Nevertheless, the novel concept of DNA barcode relying upon nucleotide divergence was not formally proposed and established for species diagnosis until 2003 by Hebert PD et al. (Hebert et al. 2003b). Since then, international initiatives have been launched across hundreds of countries to evaluate the world’s bio-diversities using this new taxonomic tool, and more than 321 K species, covering animals, plants, fungi and others, have been barcoded so far (Jeanson et al. 2011; Ratnasingham and Hebert 2007, 2013).

In light of the rapid progress and vast application of DNA barcodes, the purpose of this paper is to review these genetic markers in a variety of living organisms and provide a snapshot glimpse of mini-barcodes and DNA metabarcoding, which are essential extensions of the regular barcodes. All these barcodes, however, are heavily relying on the presence of high-quality barcode sequence reference databases that are based on good taxonomy and barcode coverage (Ratnasingham and Hebert 2007). At the end, we will also summarize some of the most exciting prospects for using this modern taxonomic tool.

## How to get a barcode

The initial motivation to have DNA barcode is to group an individual with its conspecifics using simple molecular tools instead of morphology-based procedures, which are tedious tasks requiring experienced taxonomists (Giangrande 2003). Although it has been repeatedly called into question, the core idea behind current barcodes rests on the fact that certain pieces of DNA, when aligned, can be found to vary only to a limited degree within species while this variation is much less than between species, which is referred to as the barcoding gap (Fig. 1) (Hill 2016; Liu et al. 2011; Meyer and Paulay 2005). Therefore, whether samples of target species can be differentiated largely depends on the choice of the short DNA segment. Gene regions that evolve slowly often do not differ among closely related organisms, whereas DNA sequences that evolve rapidly, on the other hand, may overwrite the traces of ancient affinities, but introduce more sequence diversity and increase the chance to distinguish between species (Cho et al. 2004; Steinke et al. 2016). In addition, the DNA section chosen must be standardized and accessible in various taxonomic groups with conservative primer binding sites so that the barcode marker technically can be robustly amplified and sequenced from a small amount of specimen through polymerase chain reaction (PCR).

Although enormous efforts have been made to find a single segment of DNA meeting all criteria outlined above, such a region has not been identified, and researchers start to realize that a single universal DNA barcode for all forms of life is unlikely to exist. This is largely because barcoding regions are not evolving neutrally since the time of speciation, and more often are influenced by weak positive or negative selection, making them suitable in some species but not others. Under such circumstances, multi-locus barcodes aiming for different living taxa have been developed and examined with respect to both their ease of amplification and their capacity to resolve species as a part of the barcode validation process (Table 1).

**Table 1:** Molecular markers routinely used for DNA barcoding studies

Organism	Region	Marker	Gene description	References
Animals	Mitochondrion	*12S*	12S ribosomal RNA	(Kocher et al. 1989; Olmstead 1996)
		*16S*	16S ribosomal RNA	(Palumbi 1991)
		*atp6*	ATP synthase F0 subunit 6	(Haag et al. 2009; Trigo et al. 2008)
		*COI*	Cytochrome c oxidase subunit I	(Hebert et al. 2003a, 2003b)
		*cytb*	Cytochrome b	(Hardman 2004; Kocher et al. 1989; Maxfield et al. 2012; Tchaicka et al. 2007)
		*D-loop*	Mitochondrial displacement loop region	(Hoelzel et al. 1991)
		*ND1*	NADH dehydrogenase subunit 1	(Thacker 2003)
		*ND2*	NADH dehydrogenase subunit 2	(Thacker 2003)
	Nucleus	*28S*	28S ribosomal RNA	(Saux et al. 2004)
		*ITS*	Internal transcribed spacer	(Smith et al. 2008)
		*Rag1*	Recombination activating 1	(López et al. 2004)
		*Rag2*	Recombination activating 2	(Hardman 2004)
		*WG*	Wingless	(Fagan-Jeffries et al. 2018)
Plants	Nucleus	*ITS*	Internal transcribed spacer	(Chen et al. 2005; Michelangeli et al. 2004)
		*ITS2*	The 2nd internal transcribed spacer	(Moorhouse-Gann et al. 2018)
	Plastid	*atpF-atpH*	Non-coding atpF-atpH intergenic spacer region	(Marcelo et al. 2010; Reginato and Michelangeli 2016)
		*matK*	Maturase K	(Fazekas et al. 2008; Parveen et al. 2012)
		*psbK-psbI*	Non-coding psbK-psbI intergenic spacer region	(Marcelo et al. 2010)
		*rbcL*	Ribulose-1,5-bisphosphate carboxylase/oxygenase large subunit	(Fazekas et al. 2008; Parveen et al. 2012)
		*rpoB*	RNA polymerase beta subunit	(Fazekas et al. 2008; Parveen et al. 2012)
		*rpoC1*	RNA polymerase beta’ subunit	(Parveen et al. 2012)
		*rps16*	Ribosomal protein S16	(Oxelman et al. 1997)
		*trnC-rpoB*	Non-coding trnC-rpoB intergenic spacer region	(Ohsako and Ohnishi 2000)
		*trnH-psbA*	Non-coding trnH-psbA intergenic spacer region	(Tate and Simpson 2003)
		*trnL (UAA)*	tRNA trnL intron	(Chen et al. 2005; Taberlet et al. 2007)
		*trnL (UAA)-trnF (GAA)*	tRNA trnL-trnF intergenic spacer region	(Sang et al. 1997)
		*trnK (UUU)*	tRNA trnK intron	(Ohsako and Ohnishi 2000)
		*ycf1*	Translocon at the inner envelope membrane of chloroplasts 214	(Dong et al. 2014)
		*ycf5*	Cytochrome c biogenesis protein CcsA	(Kress and Erickson 2007)
Fungi	Mitochondrion	*atp6*	ATP synthase F0 subunit 6	(Vialle et al. 2009)
		*COI*	Cytochrome c oxidase subunit I	(Pino-Bodas et al. 2013; Vialle et al. 2009)
		*CO3*	Cytochrome c oxidase III	(Vialle et al. 2009)
		*nad6*	NADH dehydrogenase subunit 6	(Vialle et al. 2009)
	Nucleus	*LSU*	large ribosomal subunit gene D1/D2 domains	(Eberhardt 2012; Robert et al. 2011)
		*ACT*	Actin	(Carbone and Kohn 1999)
		*TUB*	β-tubulin	(Glass and Donaldson 1995)
		*CAL*	Calmodulin	(Carbone and Kohn 1999)
		*EF1-α*	Translation elongation factor 1-alpha	(Pino-Bodas et al. 2013)
		*H3*	Histone H3	(Crous et al. 2006)
		*ITS*	internal transcribed spacer	(Vialle et al. 2009)
		*rpb2*	DNA-directed RNA polymerase II subunit	(Pino-Bodas et al. 2013)
Bacteria	Nucleoid	*16S*	16S ribosomal RNA	(Lane 1991; Sundquist et al. 2007)
		*chaperonin-60*	60 kDa chaperonin	(Brousseau et al. 2001)
		*rpoB*	RNA polymerase beta subunit	(Adékambi et al. 2009)
		*ITS*	Internal transcribed spacer	(Benga et al. 2020; Soltan Dallal et al. 2019)
Archaea	Nucleoid	*16S*	16S ribosomal RNA	(Bates et al. 2011)

### Barcodes in animals

Notwithstanding differences in evolutionary history between nuclear and mitochondrial DNA mean that a mitochondrial barcode is unlikely to be representative of nuclear divergence, in animals, regions from mitochondrial DNA (mtDNA) are preferred over nuclear genome for barcoding (Hill 2016, 2020). This is because mtDNA in most eukaryotes is known to be inherited uniparentally from the maternal parent, possessing circular DNA packaged into nucleoids without the protection of histone proteins. Despite rare recombination, mitochondrial genome, compared with nuclear DNAs, lacks sufficient DNA repair mechanisms, leading to a tenfold higher rate of nucleotide substitution in the presence of reactive oxygen species generated during the respiratory chain (Adamowicz et al. 2017). The rapid pace of sequence change in mtDNA consequently allows accumulation of differences between closely related species that have only been separated for brief periods of time.

A standard fragment of ~ 648 base pairs (bp) at the 5’ end of the mitochondrial gene coding the cytochrome c oxidase subunit 1 (*COI*), a component of an enzyme complex essential for oxidative phosphorylation, is the first and so far the most broadly adopted molecular marker for barcoding animals (Hebert et al. 2003b; Kress et al. 2005; Steinke et al. 2016). By making use of universal primers for PCR amplification, *COI* barcode has been claimed to achieve high rates of success in identification of species in test assemblages of different animal groups, mainly insects, birds and fishes (Pratheepa et al. 2014; Prum et al. 2015; Ward 2012). These PCR primers, initially described for diverse metazoan invertebrates, are fundamental to the barcode field and prevalent even today, generating informative sequences for phylogenetic analyses at the species and higher taxonomic levels (Folmer et al. 1994). Yet some studies challenged the degree of universality for *COI* and its primers for a number of reasons. For instance, the high variability of nucleotide sequences at the *COI* priming sites hinders its application to a broader spectrum of animal species (Hawlitschek et al. 2016; Shearer et al. 2002; Zangl et al. 2020). To address this issue, cocktails of degenerate primer sets were proposed for barcoding species like reptiles and amphibians (Che et al. 2012; Lyra et al. 2017; Vences et al. 2005). Moreover, *COI* barcoding region were also found to provide insufficient species resolution when it comes to organisms such as sea snails and corals because of limited nucleotide diversity (McFadden et al. 2011; Young et al. 2017). Such a shallow *COI* variation was also uncovered within many species of parasitoid wasps as the smallest interspecific divergence of only 1 bp was recorded between wasps that are known to parasitize different families of caterpillars (Smith et al. 2008). This exemplifies the integration of DNA barcoding with morphological, behavioral and ecological descriptions to improve the accuracy of species identification. In contrast, large intraspecific distance ranging from 0% to as much as 17.3% was noticed for *COI* genes in pseudoscorpions, which distorts the pattern of intra- and interspecific variation and spoils the existence of a barcode gap. This observation may result from undocumented species diversity, but also from anomalies in the COI evolution of these arachnids, indicating variable molecular change between species within different taxa (Muster et al. 2021).

Alternative barcode candidates for animals include segments from mitochondrial *cytochrome b* (cytb), *12S ribosomal RNA* (rRNA) and *16S ribosomal RNA*, and so on (Table 1) (Fernandes et al. 2020; Milan et al. 2020; Sun et al. 2019; Wong et al. 2004; Xia et al. 2012). Choices of these genetic markers are substantially due to practical reasons that a huge number of DNA sequences spanning these regions already exist in public databases before the barcoding methods became popular. Nevertheless, as a more comprehensive *COI* reference database is becoming feasible, it is argued that these alternatives may no longer perform equally well, even cases that *16S* is superior to *COI* in barcoding Arthropoda and amphibians, for example, are still reported at current stage (Sikes et al. 2017; Vences et al. 2012; Xia et al. 2012; Zangl et al. 2020).

### Barcodes in plants

As revealed in the previous section, animal mtDNAs are characterized by their rapid evolution in primary sequence, but there is a wide consensus that they are essentially invariant in gene order, especially among all vertebrates (Khan et al. 2007). However, this is not case for plants as their mtDNAs are postulated to have undergone extensive internal rearrangements, resulting in a high rate of length mutations rather than nucleotide substitution (Chevigny et al. 2020). Therefore, it is suggested that the point mutation rate in plant mtDNA is around 100 times slower than in animal mtDNA (Chevigny et al. 2020). Searching for suitable mitochondrial barcodes to delineate plant species thus has proved to be tricky and botanists thereby have focused on DNA sequences outside the mitochondrial genome.

So far, the nuclear-encoded ribosomal internal transcribed spacer (ITS) region and the chloroplast intergenic spacer *trnH-psbA* have emerged as candidates for barcoding plants, followed by others including coding sequences from plastid genes *rbcL* and *matK*, two loci now the most commonly used for plants (Kress and Erickson 2007; Loera-Sánchez et al. 2020; Yao et al. 2010). Unfortunately, no single marker from them has been found to fully satisfy all of the desired characteristics required for DNA barcodes. For instance, *rbcL* fragment is easy to amplify, sequence and align, but only yields modest discriminatory power whereas the *matK* barcode, perhaps the closest plant analogue to *COI* in animal, is difficult to amplify due to the lack of competent primers of universality (Braukmann et al. 2017; de Vere et al. 2015; Fang et al. 2019; Li et al. 2015). Even so, it is worth mentioning that the ITS2 primers designed for the second internal transcribed spacer of nuclear ribosomal DNA, which have been used successfully for a number of applications with short amplicons of 187–387 bp and addressed many of the issues, though not all, levied against the other primers (Table 1) (Moorhouse-Gann et al. 2018).

Since a standard plant barcode has been complicated by the trade-off that arises between the high variability of sequences and high conservation of primers, it is then recommended to simultaneously utilize more than one marker as a compromise that best matches the barcoding criteria (Lahaye et al. 2008). As a consequence, combinations of multiple barcode markers were shown to improve the ability to classify plants maximally by 60% when compared to a single barcode, which has persuaded researchers out of botany to take the same measures when barcoding other organisms (Group 2009; Li et al. 2021; Nitta et al. 2020; Zhang et al. 2013).

### Barcodes in microorganisms

The microorganisms discussed in this section will mainly refer to fungi, bacteria and viruses, which are all around us, having an enormous biological and economic impact, but often invisible to our naked eyes. Species discrimination is often frustrated as microorganisms only occasionally exhibit the morphological characters needed for identification in natural ecosystems. Until now, our knowledge of microbial biodiversity has been severely restricted by relying on microorganisms that can be cultured while vast majority (> 99%) cannot (Mendes et al. 2013). Luckily, PCR-based DNA barcoding techniques offer such a great opportunity to characterize microbial communities without prior cultivation.

In line with animal *COI*, the fungal counterpart is also officially recognized as an eligible barcode marker, yet it is usually excluded from consideration by mycologists due to the presence of mobile introns in the priming and sequencing regions (Seifert et al. 2007; Xu 2016; Yahr et al. 2016). Other reasons for exclusion include low nucleotide variation and a complete lack of mitochondria in some fungal linages (Wickes and Wiederhold 2018). Instead, the ITS region and the D1/D2 region of the large subunit (*LSU*) rDNA, both belonging to the nuclear ribosomal RNA genes, are the most widely used in all fungi from diverse environments (Dulla et al. 2016; Schoch et al. 2012; Scorzetti et al. 2002). These two loci can be easily amplified using relatively universal primers, and have the largest amount of reference sequence data for fungi (Blackwell 2011; Suhr and Hallen-Adams 2015). In general, ITS is better at distinguishing closely related species than *LSU*, but ITS is more difficult to align because of length differences (Takashima et al. 2019). Other fragments, like nuclear β-tubulin, translation elongation factor 1-α and calmodulin, sometimes are also applied together for selected fungal genera (Table 1), consistent with the barcoding mixtures in plants (Panelli et al. 2013; Pino-Bodas et al. 2013; Quaedvlieg et al. 2012; Robba et al. 2006).

On the other hand, *16S rRNA* gene was first advised by microbiologists as a phylogenetic tool to describe the evolutionary relationships among bacteria, archaea and eukaryotes in 1977, since when over 41 million *16S* sequences, much more than the 3 million *COI* sequences, have been deposited in GenBank (Woese and Fox 1977). However, the idea to use *16S* as the primary barcode nowadays only catches on in bacteria for a number of causes. First of all, this gene is frequently accessible in almost all bacterial species, either harmless or pathogenic. Secondly length of the gene is approximately 1500 bp, which is informative enough for analyses (Clarridge 2004). Finally, function of this gene has not changed, containing conserved sequences for universal PCR primers. Conversely, utility of *16S* is constrained in a broader taxonomic investigation by the prevalence of insertions and deletions that deeply complicate sequence alignments (Church et al. 2020; Yarza et al. 2014). Other options for barcoding bacteria include *chaperonin-60* and *RNA polymerase β subunit* (rpoB) gene, which can act as important supplementary markers to *16S* in appropriate cases (Pavan et al. 2012; Vancuren et al. 2020).

To date, detection and interpretation of virus, for example the SARS-CoV-2 responsible for the ongoing COVID-19 pandemic, is heavily dependent upon quantitative RT-PCR designed according to genomic sequences, which normally are assembled by overlapping 400 bp fragments from a serial amplicon generated in multiplexed PCR based on the latest ARTIC SARS-CoV-2 sequencing protocol (Asselah et al. 2021; Giri et al. 2021; Tyson et al. 2020; Weissleder et al. 2020). Nonetheless, viral genomes are still the most reliable source to estimate the rate of viral evolution and monitor circulating lineages. The difficulty with barcoding SARS-CoV-2 comes with the designation of target sites that are diagnostic of particular variants and, ideally, able to detect novel variants. Any attempt to capture the molecular identity of virus with standard barcoding unfortunately has turned out to be fruitless owing to the continuous introduction of new virus variants with random mutation and recombination (Bano et al. 2022). Thus, development of DNA barcode to understand viral diversity is still an open question in the field, unless multiple markers deployed to cover the whole viral genome are considered as an extension of the combination barcoding concept in plants.

## Sequence reference libraries

No matter it is conventional taxonomic approach or DNA barcoding method, the accuracy of species assignment and consequent taxonomic coverage are certainly relying on the availability of a well-curated and comprehensive reference system for judgement. A DNA barcode database is particularly vital for the latter because it fulfils the dual role of a library for data depository and a tool for monitoring the results and conclusions (Hawlitschek et al. 2016; Ratnasingham and Hebert 2007). Hence demand for high quality reference libraries has increased dramatically since the launching and extended utilization of barcoding technologies.

The Barcode of Life Data System (BOLD) is a bioinformatics platform serving for the acquisition, storage, analysis and publication of DNA barcode records (Liu et al. 2013). Core features of BOLD include open access to the entire biodiversity community, as well as the persistent linkage between a qualified barcode sequence and its source specimen with authoritative taxonomic identification. As such, BOLD workbench also implements a special analytical tool called Barcode Index Number (BIN) system, a molecular registry for codifying operational taxonomic units (OTUs) (Hausmann et al. 2013; Ratnasingham and Hebert 2013). The BIN system in principle uses well compiled algorithms and clusters similar sequences encountered in different studies into groups corresponding to presumptive species, but not necessarily actual species. Each BIN has an individual webpage displaying a unique alphanumeric identifier, nearest neighbor, all member sequences, haplotype network, specimen images, sampling map and attribution details. At this moment, the Public Data Portal of BOLD is hosting more than 715 K BINs and 9 million barcodes, which must derive from 12 million verified specimen records within the data library, highlighting a key role of morphology-based diagnostics in barcoding (Ratnasingham and Hebert 2007). All information is free for download as reference so that large amounts of data would be screened concurrently, allowing an integrated comparison of specimens identified by both molecular and morphological characters.

As soon as results are ready for public release, a copy of all sequences and crucial specimen data from BOLD would migrate to major genomics repositories worldwide, such as Genbank database at the National Center for Biotechnology Information (NCBI). Besides the *16S* and *COI* sequences mentioned earlier, a fair portion of records in GenBank actually are generated by non-barcoding studies, lack connection to a voucher specimen, and thereby may not abide to the formal barcode data standards (Sayers et al. 2020). Compared to BOLD, however, much more nucleotide sequences, including erroneous sequences uploaded by people with poor taxonomic knowledge, are currently present in GenBank, constituting a useful resource that should be closely monitored but never overlooked. Furthermore, the Basic Local Alignment Search Tool (BLAST) is attached to NCBI so that any query sequence practically can be aligned against all Genbank libraries in one go through a user-friendly web interface (Altschul et al. 1997). In contrast, selected data have to be downloaded from BOLD before blast search using local softwares or online programs. Since similarity-based alignment is a central step for classifying DNA sequences, this is why Genbank is still the best-known one-stop solution for a quick species diagnosis.

## Development of DNA barcoding

As a matter of fact, DNA barcoding, similar to any other analytical method in science, brings some controversies and concerns too, especially in the field of taxonomy, as it does not always work as effectively as first claimed (Goldstein and DeSalle 2011; Knapp et al. 2004; Miller 2007). However, in recent years, remarkable progress towards optimizing this technology has been made to improve the efficiency and lower the cost.

### Challenges to DNA barcoding

The most serious challenges in practice probably come from the initial DNA preparation and extraction, a step which is very difficult to standardize because of the complexity and diversity of the biological samples encountered, each representing different problems. It is lucky that many suspect samples, such as microorganisms that do not require prior cultivation (Sect. 2.3), can be directly boiled in reaction buffer as DNA template for PCR, while more often specimen might have been subjected to varied treatments, for instance, pH modification, high pressure, grinding or drying, which would damage DNA integrity and cause DNA degradation (Fode-Vaughan et al. 2001). Although multiple extraction methods, either in-house developed protocols or commercially available kits, are open for DNA purification on a case-by-case basis, it is still impossible to find a universal method that could be applied in all contexts and meanwhile guarantee the quality of DNA so that impurities potentially interfering downstream steps are eliminated. It should also be noticed that most DNA preparation courses conducted now are aiming to isolate genomic DNA, which could be problematic if the barcodes are targeting regions out of nucleus.

Additionally, the heteroplasmic conditions in mtDNA and the presence of nuclear pseudogenes of mitochondrial origin (numts) raise concerns as well, particularly when barcoding mitochondrial markers (D'Errico et al. 2004; Stefano et al. 2017). It is known that each eukaryotic cell contains approximately 500 to 6000 copies of mtDNA, which are tissue-specific (Friedman and Nunnari 2014). This leads to a phenomenon called heteroplasmy, where both wild-type and mutant mtDNA molecules co-exist within the same cell. The occurrence of heteroplasmic variants absolutely brings in ambiguous sequence reads, eventually influencing the accuracy of taxonomic description (Sobenin et al. 2014). To make matters worse, numts can be easily co-amplified with these mtDNA variants by using conserved PCR primers if an extraction method preferring nuclear DNA is carried out before (Guo et al. 2021). Due to the differences in genetic code between mitochondrial and nuclear genomes, numts are detected as non-functional copies of mtDNA with various sizes integrated into the nuclear chromosome naturally through unknown mechanisms. Once inserted into nucleus, numts decelerate their evolutionary rate and become molecular fossils of mtDNA, which to some extent could be indispensable for recovering ancient relationships (Mishmar et al. 2004). Nevertheless, as more eukaryotic genomes are sequenced and scanned, more numts are being discovered, which may cause misidentifications of species as numts, compared to mitochondrial barcodes, are undergoing a completely different inheritance pattern.

### Advancements of DNA barcoding

To overcome the degradation of samples with poor DNA preservation, shorter barcode regions, so-called mini-barcodes, have been developed in place of full-length barcodes over the past ten years. As an extension of DNA barcoding, mini-barcodes can be amplified with higher efficiency than regular barcodes owing to their reduced size (typically ≤ 200–300 bp), but until now they are merely considered as short versions of the full barcode markers with no real standard or reference database for mini-barcodes adopted. In addition to the deficiencies associated with normal barcoding, the rate of taxonomic discrimination is remarkably curtailed as critical information may be missed in mini-barcodes due to the length constraint (Hajibabaei and McKenna 2012; Shokralla et al. 2015). As a result, mini-barcodes cannot achieve universal application for most species unless identification at the genus or family level is warranted (Gao et al. 2019).

However, when complex samples containing DNA of different origins have to be assessed, Sanger sequencing-based barcoding protocols, either mini-barcodes or normal barcodes, will be costly and laborious, and surely produce chimeric reads with little relevance to the taxa within the sample. Then, the advent of high-throughput sequencing (HTS) technologies facilitates the emergence of DNA metabarcoding and revolutionizes our ability to barcode life. DNA metabarcoding mainly refers to the use of barcode-based (or amplicon-based) HTS for genotyping multiple species in mixtures that may take the form of propagules, or an individual organism engaging parasites, mutualists, diet items, and symbionts (Kress et al. 2015). By taking advantage of the multiplex nature of next-generation sequencing (NGS) and the third-generation sequencing platform, metabarcoding not only enables assignment of multiple species using DNA barcodes in a mixed sample and makes the data output magnitudes more reliable, but also allows simultaneous processing of DNA barcodes for thousands of diverse specimens in a single sequencing run (Coissac et al. 2012; Piper et al. 2019). Starting with minimal amounts of materials, theoretically current NGS technology with a maximum read length of 300 bp is highly suitable for mini-barcodes, while complete barcode can be recovered through assembly of short overlapping reads, or alternatively by third generation sequencing, which provides read lengths superior to any previous sequencing technology (Behjati and Tarpey 2013). A recent work with the real-time MinION sequencer, a portable third generation sequencer, has just achieved great barcode sequencing throughput at a cost of less than 10 cents, showing a promising future in this direction (Srivathsan et al. 2021). Moreover, sequences for independent gene loci can be garnered in parallel on HTS platforms in order to improve the phylogenetic resolution of generated data, a strategy first recommended in plants as reviewed in Sect. 2.2, though individual barcode from these multi-locus combinations in this context cannot be linked together without the assistance of an extensive reference library (Taberlet et al. 2012).

Consistent with the rise of DNA metabarcoding, the past few years also represent a surge in bioinformatics advancement for taxonomic analysis. Since there are already many excellent reports on computational pipelines to process large quantities of sequence reads, here we will only briefly overview the fundamentals of key steps relevant to metabarcoding. Delimiting species basically is simple to implement if reference databases pre-exist and contain information from conspecifics. In this situation, query sequences from metabarcoding studies are directly matched to identified sequences of known species in references, commonly via similarity-based and tree-based algorithms that are frequently criticized though (DeSalle and Goldstein, 2019). Conversely, if reliable reference datasets are absent, query sequences would not be linked to a taxonomic name but would be binned together to form OTUs either according to their similarity (traditionally 97%), or based on their “true” biological sequences inferred using statistical models, which are also termed exact sequence variants (ESVs) or amplicon sequence variants (ASVs) in this context (García-López et al. 2021). These biological entities next can be compared with OTUs or ASVs in different studies, such as the BIN framework introduced by BOLD, to estimate the biodiversity of target samples. Yet biological interpretation of metabarcoding data can be seriously affected by the differences between the two methods: OTUs minimize the effects of slight variations in sequences that may or may not be of interest, but a small change, as in the case of parasitoid wasps, could be capturing actual differences between species; on the contrary, ASVs are defined as all “unique reads” within a metabarcoded dataset, often leading to a wrong differentiation between the SNPs of the same species, and in the same way making sequencing or PCR errors more prominent when compared to OTUs (Molik et al. 2020). By using simulations, it has been advised that approaches utilizing ASVs outperform OTUs only when the sequencing depth is sufficient to cover a biological complexity with low polymorphisms. Otherwise conclusions drawn from OTU analyses are more consistent (Joos et al. 2020). Therefore, which method would be chosen for the bioinformatic processing of metabarcoding should be dependent on the analysis desired, although OTUs currently seem to be less preferred with the continuous improvement of sequencing technologies.

When coming back to HTS technologies, it has been argued that the current barcoding practice could soon become obsolete and irrelevant as genomic data are created by untargeted shortgun sequencing with increasing ease (Taylor and Harris 2012). In one regard, the high-throughput nature of these techniques not only allows a genomic surveillance to avoid numts, but also enables a full coverage of mitochondrial heteroplasmy to distinguish functional alleles based upon length, translation and other criteria. From another perspective, it is impossible in essence to produce a precise representation of organismal divergence using a genetic estimate taken from just parts of the genome. Taken together, it is most likely to happen that enthusiasm for DNA barcode to the end will transition to a larger endeavor of archiving accessible genomic data. Before that, however, more sophisticated bioinformatic modellings with user-friendly interfaces, as well as huge genome storages as references with information dedicated to taxonomic relatedness must be developed. At present, the question lies in whether the barcoding enterprise tends to take measures and evolve its methodologies to embrace novel techniques that are inevitably on the way? In fact, many botanists have conducted genome skimming for entire plastid genomes and nuclear ribosomal regions to cover all of the different standard plant barcoding regions as an extended barcode, while others assemble the whole organelle genomes as a resource for validating and designing short, informative barcode markers with diagnostic nucleotides (Coissac et al. 2016; Kreuzer et al. 2019). These attempts represent a stepping stone on the continuum between routine barcode movements and complete genome sequences. Along this path, we believe DNA barcoding will be capable of further exploring its potential and opportunities, and perhaps one day will encompass other “omics” approaches such as proteomics and metabolomics.

## Practical utilities of DNA barcoding

Undoubtedly, DNA barcoding is a chief component of the modern diagnostic toolbox with increasing applications in taxonomy, systems biology and ecological studies. Prior to barcoding, conventional approaches for classification of species mainly rely upon the characterization of distinguishable morphology while many organisms exhibit morphologically distinct stages controlled by gender or life cycles (Hall and Martín-Vega 2019). Furthermore, suspect specimens may be damaged or incomplete with only part of tissue feasible for identification. All these pitfalls would render morphological determination unclear or unlikely, but can be easily avoided with molecular barcoding. Besides traditional way by sampling separate individuals, barcode technology, especially metabarcoding, can be adopted for assessment to dietary items using gut contents and scats of animals, or utilized for analyzing environmental samples, namely samples from soil, water and even air that possibly contain DNA materials from life, for biomonitoring and disease screening (Chaves et al. 2012; Haag et al. 2009; Staats et al. 2016). Together with mini-barcodes, it could further mitigate problems with fragmented DNA present in the environment, gut contents or other sources of exogenous DNA (Prerna Vohra 2013). Another potentially valuable utility of combining metabarcoding with mini-barcodes is to analyze invertebrate-derived DNA (iDNA), where vertebrate genetic material is extracted from diverse invertebrates, including terrestrial leeches, mosquitoes, midges, blow flies and ticks (Schnell et al. 2015). iDNA has recently been proposed as a powerful non-invasive tool to detect vertebrate species and to survey their population as long as the information regarding the biology, habitats, behaviors and diets of relevant invertebrates is secured.

**Fig. 2:**
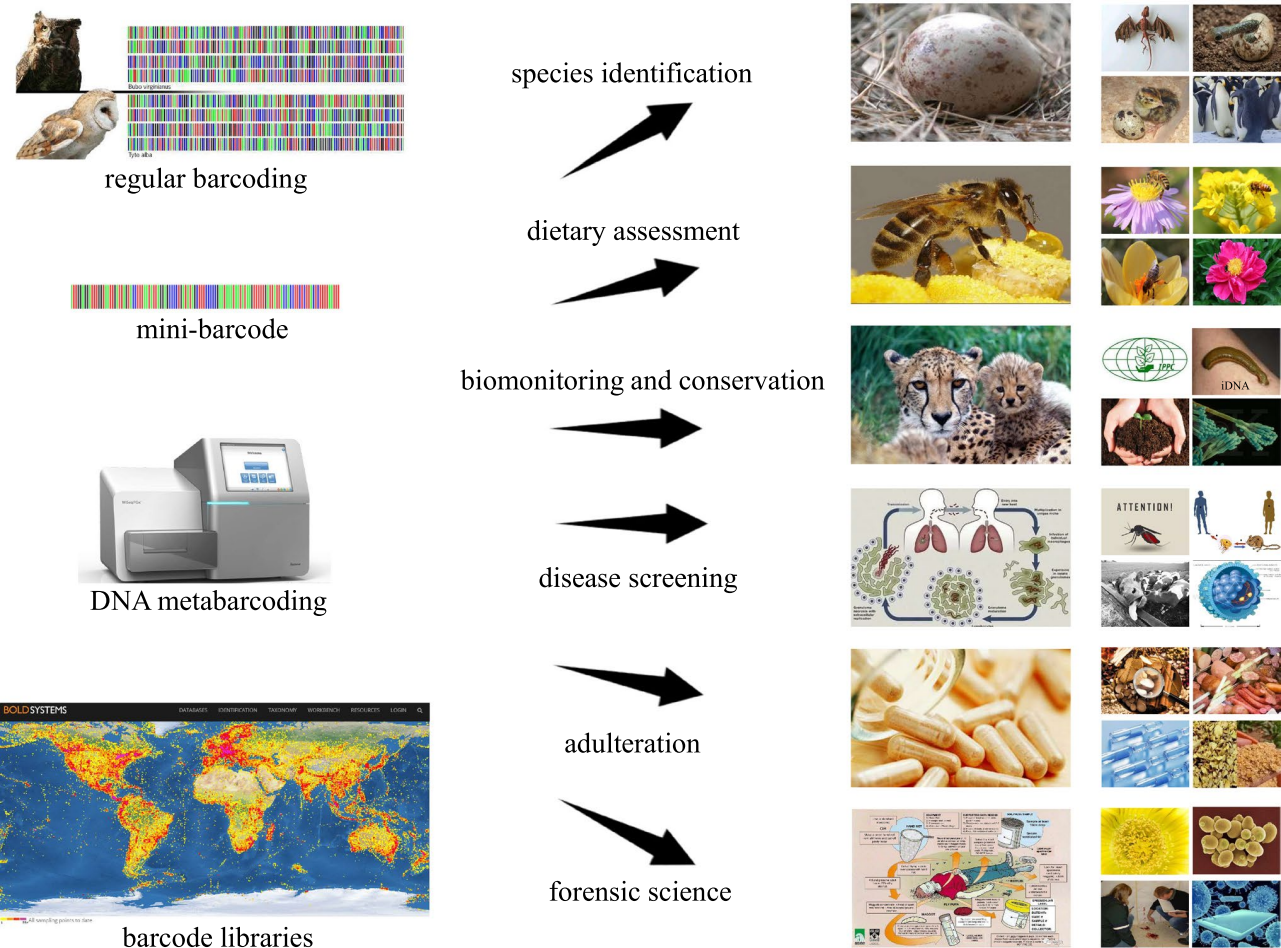
Practical utilities of DNA barcoding technology

The usefulness of DNA barcoding is not restricted to scientific research of biodiversity, but also concerns conservation, public health and biosafety (Fig. 2). It is not surprising that barcoding is highly desirable for customs and national authorities in the conservation area of rare wildlife. International conventions such as the Convention on International Trade in Endangered Species of Fauna and Flora (CITES) have categorized more than 35,000 species as threatened by extinction (Wyatt 2020). And DNA barcodes have been demonstrated to be helpful to monitor illegal collection and trade of protected species and their products when morphological characters were equivocal (Chapagain et al. 2021). Additionally, the risks of pandemic spillover are higher than ever with increasingly intimate associations between humans and wildlife (or their meat), some of which might serve as hosts or vectors for medically important pathogens. For example, there are about 3500 species of mosquitoes, but only a handful of species spread malaria, dengue fever and other diseases in tropical areas (James 2007; Lee et al. 2018). DNA barcoding actually has been reported to successfully determine mosquitoes involved in disease transmission and public health. In the same way, genetic authentications using barcodes are also becoming more and more common in food adulteration and manufacture of drugs of natural origin (e.g. herbal products or mixtures in traditional Chinese medicine), misidentification of which sometimes could be poisonous and life-threatening (Kreuzer et al. 2019; Wu et al. 2019). Aside from herbal medicine, metabarcoding of pollen and fungal spores can also be incorporated into forensic palynology and security intelligence to link persons or objects with particular places and times, given pollen and fungal spores’ ubiquity in the environment, their potential for geographic and temporal inference, and their long-term durability (Bell et al. 2016).

## Conclusions

By employing sequence divergence in several short and standardized gene fragments, DNA barcode and its library have become an invaluable addition to our suite of tools to understand life and nature. Although posing many controversies, DNA barcoding no doubt holds great promise for potentially widespread scientific and practical benefits. With the exploration of mini-barcodes and metabarcoding in DNA-based species delineation, it is believed that barcode techniques will be further integrated into a wider context of scientific, political, economic and social areas.

Then, as the barcoded reference species expands across the tree of life, ultimately one must ask whether it is possible to barcode all life on Earth. In theory, the barcoding process is able to yield 100% accuracy of species delimitation as long as robust thresholds defining species boundaries are established, which is truly tough to settle for all of life (Matute and Sepúlveda 2019). Also, it would be naïve to portray a species or infer a phylogeny without any corroborating evidence other than certain pieces of DNA sequences. In the absence of other evidence, DNA barcoding creates hypotheses regarding new species rather than outright discovering them (Taylor and Harris 2012). More importantly, it should be noted that barcoding must supplement morphological data for species description, which usually fails to break into the mainstream of barcoding studies despite the fact that morphological identification laid the foundation of all barcode databases. To sum up, what we know today is that no single classification approach can be applied universally for all species. DNA barcodes in conjunction with traditional taxonomic tools for sure are more rapid and more reliable than any method alone for disclosing cryptic and overlooked biodiversity.
